# Several wall-associated kinases participate positively and negatively in basal defense against rice blast fungus

**DOI:** 10.1186/s12870-016-0711-x

**Published:** 2016-01-16

**Authors:** Amandine Delteil, Enrico Gobbato, Bastien Cayrol, Joan Estevan, Corinne Michel-Romiti, Anne Dievart, Thomas Kroj, J.-B. Morel

**Affiliations:** CIRAD, UMR BGPI INRA/CIRAD/SupAgro, Campus International de Baillarguet, TA A 54/K, 34398 Montpellier, France; INRA, UMR BGPI INRA/CIRAD/SupAgro, Campus International de Baillarguet, TA A 54/K, 34398 Montpellier, France; CIRAD, UMR DAP INRA/CIRAD/SupAgro, Avenue Agropolis, 34398 Montpellier Cedex 5, France

**Keywords:** Rice, Wall-associated kinase (WAK), Basal immunity, Blast fungus

## Abstract

**Background:**

Receptor-like kinases are well-known to play key roles in disease resistance. Among them, the Wall-associated kinases (WAKs) have been shown to be positive regulators of fungal disease resistance in several plant species. *WAK* genes are often transcriptionally regulated during infection but the pathways involved in this regulation are not known. In rice, the *OsWAK* gene family is significantly amplified compared to Arabidopsis. The possibility that several *WAKs* participate in different ways to basal defense has not been addressed. Moreover, the direct requirement of rice *OSWAK* genes in regulating defense has not been explored.

**Results:**

Here we show using rice (*Oryza sativa*) loss-of-function mutants of four selected *OsWAK* genes, that individual OsWAKs are required for quantitative resistance to the rice blast fungus, *Magnaporthe oryz*ae. While OsWAK14, OsWAK91 and OsWAK92 positively regulate quantitative resistance, OsWAK112d is a negative regulator of blast resistance. In addition, we show that the very early transcriptional regulation of the rice *OsWAK* genes is triggered by chitin and is partially under the control of the chitin receptor CEBiP. Finally, we show that OsWAK91 is required for H_2_O_2_ production and sufficient to enhance defense gene expression during infection.

**Conclusions:**

We conclude that the rice *OsWAK* genes studied are part of basal defense response, potentially mediated by chitin from fungal cell walls. This work also shows that some *OsWAKs*, like *OsWAK112d*, may act as negative regulators of disease resistance.

**Electronic supplementary material:**

The online version of this article (doi:10.1186/s12870-016-0711-x) contains supplementary material, which is available to authorized users.

## Background

Plants have evolved the ability to detect potentially pathogenic microorganisms via pattern-recognition receptors (PRRs) localized on the surface of plant cells [1]. PRR proteins recognize Pathogen Associated Molecular Patterns (PAMPs) that are conserved motifs in the pathogen and Damage Associated Molecular Patterns (DAMPs) that derive from the damages caused by pathogen ingress [2]. Detection of pathogen through PRRs triggers PAMP-triggered immunity (PTI, also called basal defense) which is accompanied with rapid production of reactive oxygen species (ROS), activation of mitogen-activated protein kinases (MAPKs) and changes in expression of immune-related genes [2].

So far eight bacterial, four fungal PAMPs and 20 PRRs have been identified molecularly [3]. The best studied PAMP recognition systems in plants are represented by the bacterial flagellin recognized by the *Arabidopsis thaliana* FLS2 receptor and the fungal chitin recognized by the CEBiP receptor [1]. The FLS2 protein belongs to the Receptor-like Kinase (RLK) gene family. The typical structure of an RLK is an extracellular receptor domain that recognizes the PAMP molecule, a transmembrane domain and an intracellular kinase domain [4]. The CEBiP protein is composed of an extra-cellular LysM domain anchored to the membrane but does not contain any kinase domain [5]. FLS2 and CEBiP are found associated with RLK proteins like BAK1 in Arabidopsis and CERK1 in rice respectively [1]. FLS2 and CERK1 are positive regulators of basal defense since mutations in these genes lead to a decrease of resistance in Arabidopsis [6, 7] or to a decrease of basal defense in rice [8]. By contrast to PAMP, our knowledge on DAMP detection is much less advanced and only three pairs of PRRs and DAMP have been identified so far [3]. One of these is the PRR/DAMP pair between the Arabidopsis Wall-Associated Kinase 1 (AtWAK1) and oligogalacturonides (OGs) [9] derived from the pectin embedded in the cell-wall of most plants [10].

Wall-Associated Kinases are characterized by an extracellular domain composed of one or several repeats of the Epidermal Growth Factor (EGF) domain. The EGF domain is known in animals to bind a very large range of small peptides and to dimerize upon calcium binding [11]. EGF- containing proteins can form homo and heterodimers after ligand binding in animals [12]. Based on homology with the kinase domain of five WAKs from Arabidopsis [13], 21 genes coding WAK-like (WAKL) proteins were identified in Arabidopsis and 125 in rice, revealing an expansion of the WAK family in monocots [14, 15]. For simplicity and following previous nomenclature in rice [15], the WAK-like proteins are referred as WAKs. Among the rice WAKs, 67 have a *bona fide* EGF extracellular domain. Only a few WAKs from Arabidopsis or rice have been shown to possess kinase activity [16, 17]. Similarly, only a few WAKs have been localized to the plasma membrane in Arabidopsis [18] or rice (OsWAK1) [17], (OsDEES1/OsWAK91) [19]. More recently, maize ZmWAK was shown to be localized to the plasma membrane [20]. Moreover, WAKs seem to be found in large membrane protein complexes of unknown composition [21]. It is not known whether WAKs associate with other RLKs to ensure appropriate function like several other RLKs [22].

In plants, several ligands were shown to bind the extracellular domain of WAK proteins. For example the AtGRP3 protein binds to AtWAK1 [21] and pectin and OGs bind AtWAK1 and AtWAK2 [23–25]. It was shown that upon pectin treatment AtWAK2 activates the mitogen-activated kinases MPK3 and MPK6 and that a TAP-tagged (Tandem Affinity Purification) version of AtWAK2 constitutively activates ROS production and defense gene expression [26]. However, there is no indication that native WAKs can trigger ROS and there is only very limited information on defense gene expression during infection [20].

WAKs are involved in plant development [27]. For instance, AtWAK1 and AtWAK2 are required for cell wall expansion [28]. Accordingly, WAK mutants are often affected in their development. In rice, plants silenced for *OsDEES1*/*OsWAK91* displayed fertility deficiency [19] that was attributed to a defect in embryo development. Plants silenced for the rice indica *OsiWAK1* gene were stunted [29] and in Arabidopsis, silencing of *AtWAK1* and *AtWAK2* is lethal [28].

The role of WAKs in plant disease resistance initially came from indirect evidence with WAK mutants affected in the triggering of defense-related response [18]. Later, several studies provided direct evidence that *WAK* genes participate to resistance. First, it was shown that the *RFO1/WAKL22* gene is responsible for quantitative resistance to *Fusarium* [30] and *Verticilium* [31]. More recently, two distinct wall-associated kinases from maize were shown to be responsible for a major QTL for resistance to the soil-borne fungus *Sporisorium reilianum* (*ZmWAK*) [20] and one against the foliar fungal pathogen *Exserohilum turcicum* (*Htn*) [32]. Secondly, several mutant analyses of *WAK* genes provided evidence for their involvement in disease resistance. The over-expression of *AtWAK1* led to enhanced resistance to *Botrytis* [9] and over-expression of *OsWAK1* enhanced resistance to *Magnaporthe oryzae* [17]. On the other hand, silencing of *SlWAK1* in tomato lead to enhanced susceptibility to the bacterial pathogen *Pseudomonas synringae* pv *tomato* [33]. Other examples of the effect of WAKs on bacterial and fungal resistance are reported although the corresponding proteins miss an EGF domain (*OsWAK25*) [34] or a kinase domain (At5g50290) [35]. Thus several WAK mutants seem to act as positive regulators of disease resistance to fungi and bacteria without visible developmental phenotypes. However, there is thus far no indication that PTI is affected in these mutants.

Another indication that WAKs are related to disease response comes from the observation that *WAK* genes are often regulated by bacterial infection in Arabidopsis [33] and by blast infection in rice [36, 37]. Quite interestingly, there are two cases of pathogens that manipulate WAK gene expression by either expressing small RNA interfering with their RNA [35] or by an unknown mechanism [33]. Thus WAKs are important components of basal defense that pathogens try to inhibit. PAMPs can also directly regulate the expression of *WAK* genes [38]. Flagellin induces several *WAK* genes in Arabidopsis [39] and tomato [33]. Chitin induces *OsWAK91* in rice in a CEBiP dependent manner in cell cultures [5] and the *AtWAKL10* gene in Arabidopsis [40]. However, the global regulation of *WAK* genes in PTI is not well understood.

Here we report that several rice *WAK* genes are up-regulated while *OsWAK112d* is down-regulated by fungal infection in rice. Part of this transcriptional control is likely due to chitin detection by the chitin receptor CEBiP. We provide evidence that OsWAK14, OsWAK91 and OsWAK92 act as positive regulators of quantitative resistance, while OsWAK112 acts as a negative regulator. By studying *OsWAK91* mutants, we demonstrate that this WAK significantly participates to ROS production and defense gene expression during infection.

## Results

### *OsWAK* expression is influenced by blast infection

Previous transcriptome analysis identified five *OsWAK* genes differentially expressed upon infection by *M. oryzae* in rice (Additional file 1). Phylogenetic analysis revealed that excluding OsWAK1, all blast responsive WAKs are from one major clade of rice WAKs designated WAKb and that they belong to four different, clearly distinct WAKb sub clades (Additional file 2).

**Fig. 1:**
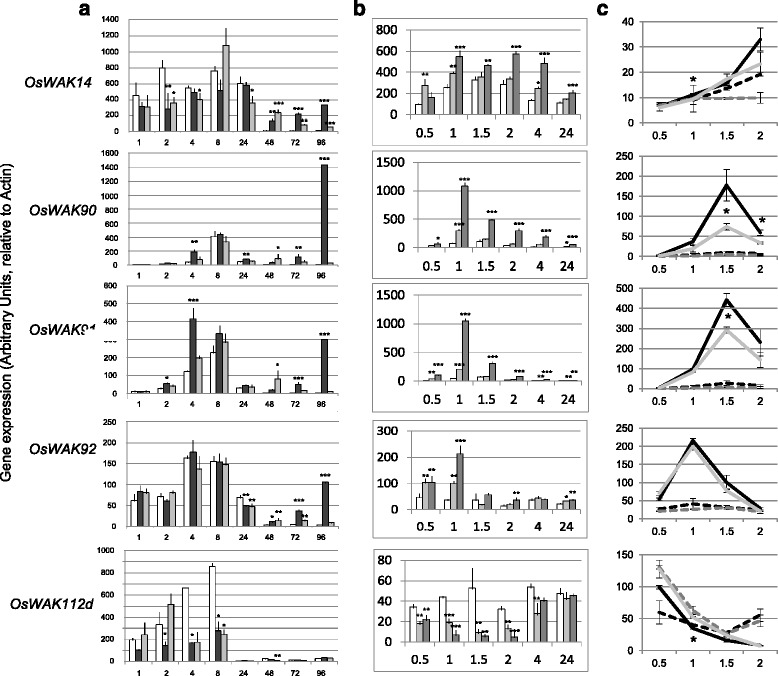
*OsWAK* gene expression during infection and upon chitin treatment. *WAK* gene expression was measured by quantitative RT-PCR in leaf tissues under inoculation by *M. oryzae* (**a**), after chitin treatment (**b**) and in the *cebip* mutant (**c**). The data were normalized using Actin and all values shown are expressed as Arbitrary Units. For *OsWAK112d*, the two alternative transcripts described (Additional file 3A) gave the same expression pattern and the longest one is shown. Mean values are provided with the standard error (*n* = 4). Statistical differences were evaluated according to one-way ANOVA followed by Dunnett’s test relative to Mock condition for each data point *P* <0.05 (*), *P* <0.01 (**) and *P* <0.001 (***). For panel (**c**), only significant tests between wild-type and *cebip* mutants treated with chitin are shown. **a** Plants inoculated with gelatin only (Mock treatment: *white bars*) or with *Magnaporthe oryzae* (virulent isolate FR13: *dark bars*; avirulent isolate CL367: *grey bars*) at different hours after treatment. **b** Chitin and water were sprayed on rice plants. The values are the mean calculated from four independent biological replicates (*white bars*: mock; *light grey bars*: 100 μg/mL chitin; *dark grey* bars: 1000 μg/mL chitin). **c** Regulation of *OsWAK* genes after chitin treatment (*continous lines*, 1000 μg/mL chitin) or mock-treated (*dashed lines*) in the *cebip* mutant (*grey lines*) and the corresponding null-segregant (wild-type) plants (*black lines*)

To further investigate on these blast-responsive WAKs, their expression profile in compatible and incompatible interactions was measured at early and late infection stages (1 to 24 h post-infection (hpi) and 48 to 96 hpi) using isolates FR13 and CL367 respectively (Fig. 1a). All *OsWAKs* transcripts were differentially expressed during infection in at least one time point and expression changes were similar in compatible and incompatible interactions. At late infection stages, except *OsWAK112d*, all *OsWAK* genes were induced and expression induction was often more evident in susceptible plants than in resistant ones. During early infection (2 and 4 hpi), the expression of *OsWAK90* and *OsWAK91* was induced. By contrast, *OsWAK112d* transcripts, and to a lower extent *OsWAK14,* were repressed early. Thus, among the various transcriptional changes found for the tested *OsWAKs*, most were induced during infection, sometimes even before fungal penetration (< 24hpi) and one, *OsWAK112d* was repressed.

### Chitin triggers *OsWAK* gene expression

The early and non-isolate specific differential expression of the *OsWAK* genes (Fig. 1b) suggested that a PAMP common to these isolates was the trigger for *OsWAK* gene regulation during early infection. Chitin is common to all fungi and has been shown to act as an important PAMP in several biological systems [7, 41] including rice [5, 42]. To test the effect of chitin on *OsWAK* gene expression, plants were sprayed with chitin oligomers and the expression of *OsWAKs* was determined. Chitin strongly and rapidly induced the expression of the blast-induced genes (Fig. 1b) *OsWAK91* and *OsWAK90* (almost 20 fold induction of both genes after 1 h) while the blast-repressed *OsWAK112d* was down-regulated (8-fold) by the chitin treatment. The expression of *OsWAK14* and *OsWAK92* was induced to a much lower extent by the chitin treatment. Therefore, we conclude that *OsWAK* genes show similar expression trends after chitin treatment (Fig. 1b) and during early stages of blast infection (Fig. 1a).

In order to test whether chitin regulates *OsWAKs* in a receptor-dependent manner, *OsWAK* gene expression was analyzed in mutant lines deficient for CEBiP, the major chitin receptor in rice [5, 42, 43]. Chitin oligomers were sprayed on *cebip* loss-of-function mutant plants [44] and gene expression was measured until 2 h after treatment (Fig. 1c). Mutants in *CEBiP* have been shown to display a reduced transcriptional response of *OsWAKs* to chitin oligomers [43]. For *OsWAK90* and *OsWAK91,* chitin-triggered gene expression was significantly reduced in the *cebip* mutant (Fig. 1c). By contrast the induction of the other chitin-responsive *OsWAK* genes and the repression of *OsWAK112d* were only slightly affected by *cebip* mutation. This supports our hypothesis that the CEBiP receptor is required for proper activation and repression of several *OsWAK* genes upon chitin treatment.

### Different requirements of *OsWAKs* for quantitative resistance to rice blast

To elucidate the role of blast- and -chitin responsive *WAKs* in disease resistance, mutant lines were searched in the OryzaTagLine mutant collection [45, 46]. Two allelic lines for *OsWAK14* (*wak14-1* and *wak14-2*) and one line for each *OsWAK91*, *OsWAK92* and *OsWAK112d* were identified (Additional file 3A). The insertion lines harboured a *Tos17* retrotransposon inserted into the coding sequence of the respective *OsWAK* genes. For each insertion line, we isolated one homozygous line for the *Tos17* element (mutant) and one sister line without the *Tos17* element (later called null-segregant: NS). We confirmed that the expression of the targeted *OsWAK* gene was reduced in each mutant line as compared to the null-segregant line (Additional file 3B). The mutant lines did not show any obvious growth phenotype (data not shown), including full fertility in the *wak91* mutant despite previous report showing that RNAi of this gene leads to sterility [19].

To determine whether the *wak* mutations could affect *R* gene mediated resistance, we tested the avirulent *M. oryzae* isolate CL367 on *wak* mutant and null-segregant lines. After inoculation with isolate CL367, we did not observe any difference between the *wak* mutants and their respective null-segregant (data not shown).

**Fig. 2:**
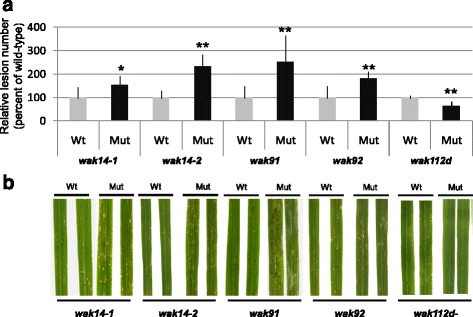
Resistance to *M. oryzae* is affected in *wak* loss-of-function mutants. Plants were inoculated with the virulent isolate FR13 of *M. oryzae* and disease was measured 7 dpi. For each line, a homozygous mutant (Mut) and the corresponding null-segregant (Wt) is presented. **a** The number of lesions on the youngest fully expanded leaves of at least 7 plants of homozygous and null-segregant lines was counted 7 dpi. The values were transformed in percentage relative to the mean of null-segregant lines, and means and standard deviations were calculated. A t-test was performed to establish whether one given mutant line was different from its corresponding null-segregant line (*: *p* <0.05; **: *p* <0.001). The experiment was repeated seven times and one representative experiment is shown. **b** Pictures were taken 5 days post inoculation. Susceptibility is characterized by the *grayish lesions* while *small brown spots* derive from some residual resistance

To test the impact of WAK mutations on basal resistance, we inoculated the *wak* mutants with the virulent *M. oryzae* isolate FR13. The *wak14-1*, *wak14-2*, *wak91* and *wak92* mutants were all more susceptible to isolate FR13 than their respective null-segregant controls (Fig. 2a, b) and displayed an increased number of sporulating lesions (1.6-fold more for *wak14-1*, 2.3-fold for *wak14-2*, 2.5-fold for *wak91* and 1.8-fold for *wak92*). On the opposite, *wak112d* mutant plants were more resistant to blast disease. This was manifested by a 1.6-fold reduction of disease lesion numbers. Thus *wak* mutants are affected for blast susceptibility, suggesting that the corresponding *OsWAK* genes are important elements of basal disease resistance.

### Effects of over-expression of the *OsWAK91* and *OsWAK112d* genes on basal resistance to blast fungus

In order to further investigate the role of the *OsWAKs* in blast resistance, we decided to produce rice plants that over-express *OsWAK91* and *OsWAK112d*. We focused on these two genes as they represented the most pronounced expression patterns after infection (Fig. 1) as well as the strongest disease phenotypes in the corresponding loss-of-function mutants (Fig. 2).

**Fig. 3:**
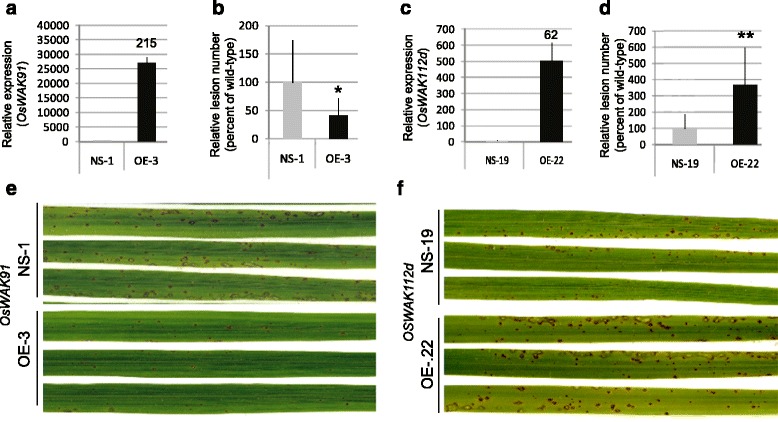
*OsWAK91* and *OsWAK112d* over-expresser lines are affected for *M. oryzae* resistance. Expression of *OsWAK91* and *OsWAK112d* was measured by quantitative RT-PCR in non-infected lines over-expressing (OE) *OsWAK91* (**a**) or *OsWAK112d* (**b**) genes and the corresponding null-segregant lines (NS). Gene expression, calculated from three biological replicates, was normalized using Actin. The fold change between NS and OE is also indicated (**a** and **b**). Plants were inoculated with the virulent isolate GY11 of *M. oryzae.* The total number of lesions was counted as in Fig. 2 in the lines OE-*OsWAK91* (**c**) or OE-*OsWAK112d* (**d**). For each mutant line, the average number of lesions over more than 7 plants was calculated for the corresponding over-expresser and null-segregant line. This value was used to calculate the percentage of lesions per individual mutant plant as compared to the mean of the null-segregant plants. The mean and standard deviation was then calculated. This experiment was repeated three times and one representative experiment is shown. A t-test was done to evaluate the significance of the observed differences (*: *p* <0.05; **: *p* <0.001). Pictures of typical symptoms on over-expresser and null-segregant lines were taken 5 days post inoculation (**e** and **f**). Several other over-expresser lines were also produced and analyzed (Additional file 4)

After infection with the virulent strain FR13, all 10T0 plants over-expressing *OsWAK91* showed reduced symptoms compared to plants transformed with the empty vector (Additional file 4A, B). By contrast, over-expression of the *OsWAK112d* gene increased susceptibility compared to empty vector (Additional file 4C, D). In order to further analyze these disease resistance phenotypes, single locus insertion lines were selected. In the T1 generation, plants without T-DNA and sister plants with T-DNA were identified and their seed amplified to give rise respectively to homozygous null-segregants (NS) and over-expresser lines (OE) (Fig. 3a, b). The OE and NS lines were inoculated with the moderately virulent *M. oryzae* isolate GY11. We observed a decrease in lesion number for the OE-*WAK91* plants (60 %; Fig. 3c, e) and an increase for the OE-*WAK112d* plants (264 %; Fig. 3d, f). Thus, it appears that the over-expression of *OsWAK91* or *OsWAK112d* alters basal resistance and that both genes have opposite effects on quantitative resistance, consistent with the phenotypes observed with the loss-of-function mutants.

### Defense induction in *OsWAK91* mutant lines

To further characterize the increased partial resistance of OE-*WAK91* lines, individual interaction sites were examined by microscopy (Additional file 5). Two days after infection, the number of hyphae that penetrated into rice epidermal cells was only slightly reduced in OE-*WAK91* lines and 3 days after inoculation, the proportion of fungal hyphae that invaded more than one cell was strongly reduced. These data suggest that *OsWAK91* over-expression enhances resistance against fungal penetration and affects the *in planta* growth of the blast fungus and/or the invasion of new cells.

**Fig. 4:**
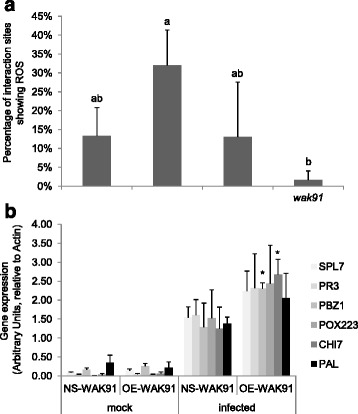
Defense gene expression and H_2_O_2_ production in OE-*WAK91* lines. OE-WAK91 lines were inoculated with *M. oryzae* (GY11 moderately virulent isolate) and we measured two facets of plant defense response at 48 hpi: H_2_O_2_ production (**a**) and expression of genes responsive to infection (**b**). Mean values are provided with the standard error (*n* = 4). Statistical differences were evaluated according to one-way ANOVA followed by Fischer’s (**a**) or Dunnett’s (**b**) tests: P < 0.05 (*), *P* <0.01 (**) and *P* <0.001 (***). **a** H_2_O_2_ production in loss-of function and OE-*OsWAK*91 was expressed as the percentage of infection sites (>100 counted for each genotype) showing DAB staining. **b** Expression of defense-related genes in the *OsWAK91* over-expresser (OE) and null-segregant (NS) lines

To get further insights on how *OsWAK91* gene affects basal resistance, we measured several molecular markers of basal defense in the *OsWAK91* lines (Fig. 4) two days post inoculation, at a time where only small differences in fungal growth were visible (Additional file 5). We first tested whether the oxidative burst, one of the earliest responses to fungal invasion was affected. Two days after inoculation, the production of H_2_O_2_ as measured by DAB staining was two times higher in OE-*WAK91* lines and two times lower in ko-*WAK91* lines compared to their respective controls (Fig. 4a). Using qRT-PCR on defense marker genes, we then evaluated whether defense was enhanced before and during infection. We could not detect any significant difference of expression of the defense marker genes before inoculation in OE-*WAK91* compared to control lines (Fig. 4b). By contrast, several markers tested were significantly induced to higher levels in OE-*WAK91* plants 48 h post inoculation (Fig. 4b). Thus, we could correlate the increased level of resistance of OE-*WAK91* visible at 3 dpi with an increase in H_2_O_2_ production and defense-gene induction at 2 dpi.

## Discussion

### The expression of *OsWAK* genes is induced by chitin under the control of the CEBiP receptor

Several previous reports indicate that *OsWAKs* genes are transcriptionnaly regulated during *M. oryzae* infection [36]. By investigating the regulation of *OsWAKs* during early infection steps, before penetration of *M. oryzae*, in both resistant and susceptible plants we confirmed that five selected *OsWAK* genes are differentially expressed upon infection by virulent and avirulent isolates of *M. oryzae* (Fig. 1a). We showed that the differential expression of the *OsWAK* genes was, overall, more intense in the case of quantitative resistance (compatible interaction with FR13 isolate) than in complete resistance (incompatible interaction with CL367 isolate). This is contrasting with the report that the *OsWAK1* gene is more induced in resistant plants than in susceptible plants [17]. Quite interestingly, the *OsWAK1*gene belongs to another phylogenetic group than all *OsWAKs* tested here (Additional file 2). This may explain these differences in transcriptional regulation.

The *OsWAK91* and *OsWAK112d* genes were characterized by their differential expression early after inoculation (2 hpi, before penetration). This is also contrasting with the observation that the *OsWAK1* gene is induced rather late after infection (16–24 hpi) [17]. Quite notably, the *OsWAK112d* gene was down-regulated in the early phases of infection (Fig. 1a) and as expected from Vergne et al. [37] was slightly induced after 24 hpi. The triggering of some of *OsWAKs* expression before fungal penetration (< 24 hpi) suggested that a molecule constitutively present in fungi could elicit this expression. Chitin, a molecule that is known to elicit defenses in rice [5] was tested. Chitin alone triggered induction of the *OsWAK90* and *OsWAK91* genes and the repression of the *OsWAK112d* gene (Fig. 1b), thus mimicking the events observed during infection (Fig. 1a). Using the *cebip* mutant, we show that the regulation of expression by chitin of *OsWAK90* and *OsWAK91*, and to a lower extent the other *OsWAK* tested, is controlled by CEBiP. Thus *OsWAK* gene regulation by chitin is partially affected by the *cebip* mutation. Testing CERK1 [8], LYP4 and LYP6 [47] mutants is required to establish if these other chitin receptors are involved in triggering *OsWAKs* expression. *AtWAK1* and *AtWAK2* genes are slightly down-regulated by flg22 [9, 38] while *AtWAKL* genes are up-regulated [39]. Similarly, transcriptome analysis indicates that the expression of the *AtWAKL10* gene is triggered by chitin [40]. Thus *WAK* gene regulation by PAMPs seems to be a common feature in plants.

### *OsWAK* genes are required for quantitative resistance to rice blast fungus

There are now six reports that *WAK* genes are involved in disease resistance. In Dicots, several studies report on the role of WAKs in disease resistance: *RFO1/WAKL22* [30, 31], *AtWAK1* [9], *SlWAK1* [33]. In rice, there is only one report that the over-expression of the *OsWAK1* gene enhanced resistance to *M. oryzae* [17]. More recently two QTL for resistance to fungal disease in maize were shown to encode *WAK* genes [20, 32]. Our work showing the involvement of four *OsWAK* genes in rice blast resistance significantly extends this list and reinforces the notion that these receptor-like kinases play a central role in basal resistance. Indeed, we show that three loss-of-function mutants in *OsWAK14*, *OsWAK91* and *OsWAK92* have reduced basal resistance towards a virulent isolate of the rice blast fungus (Fig. 2). The two independent mutant alleles of the *OsWAK14* gene displayed similar phenotypes, suggesting, despite the lack of complementation analysis, that *OsWAK14* is a positive regulator of blast resistance. The fact that *OsWAK91* loss-of-function displayed reduced resistance combined to the fact that over-expression of the *OsWAK91* gene leads to enhanced resistance (Fig. 3a, c, e) suggests that this gene is also a positive regulator of blast resistance. Whether *OsWAK92* is also a positive regulator of basal resistance awaits additional genetic demonstration.

Quite strikingly, the mutant in the *OsWAK112d* gene and over-expression of the *OsWAK112d* gene led to increased resistance and susceptibility respectively. Altogether with the observation that this gene is repressed upon infection (Fig. 1a), this indicates that *OsWAK112d* is a negative regulator of basal resistance that rice plants repress during infection. Similarly, the Arabidopsis LRR-RLK *FERONIA* gene was shown to negatively regulate the signaling pathway involved in basal defense [48]. Thus our results extend the observation that negative regulation by RLK of signaling pathways could be a common trend in plants.

Thus far the kinase domains of the majority of RLK involved in disease resistance belong non-RD group [4], including *OsWAK1*. It is noteworthy that all *OsWAKs* tested in this study belong to the WAKb sub-group (Additional file 2) which are characterized by kinase domains of the RD type [49], like all known Arabidopsis WAKs. Thus this work indicates that several *OsWAK* genes from this WAKb/RD kinase sub-group are also regulators of quantitative resistance to the blast fungus in rice.

### *OsWAK91* participates in plant defense response

Measurement of H_2_O_2_ production (Fig. 4a) and defense genes expression (Fig. 4b) in *OsWAK91* mutant lines indicated that this gene is involved in both responses to pathogen. This is consistent with the enhanced basal resistance levels observed in over-expressing plants (Fig. 3a, c, e). In particular plants over-expressing *OsWAK91* displayed enhanced H_2_O_2_ production and defense-related gene expression. Only limited evidence suggests that WAKs can regulate, directly or indirectly, defense-gene expression. Indeed, in their study, Zuo et al. [20] provided some evidence that three genes (*NPR1*, *PR5* and *LOX1*) out of six tested were slightly up-regulated by ZmWAK upon infection. Our results are consistent with such slight modifications. By contrast to Zuo et al. [20], we did not observe any enhanced expression of defense-marker genes before inoculation triggered by the over-expression of *OsWAK91*. Thus WAKs may have different ways for activating defense.

As proposed by others [33], our data support a model in which recognition of PAMPs like chitin would lead to an increased expression of *OsWAKs* and an increase in OsWAK receptors at the plasma membrane. Subsequently, DAMPs produced by pathogen ingress could be recognized by OsWAK91, triggering an enhanced immune response. Further experiments are needed to test this model, such as extensive time course analysis of defense response in *wak91* mutants.

## Conclusions

We have shown that several *OsWAK* genes are required for quantitative resistance in rice to the blast fungus *M. oryzae*. More importantly, we showed that among the four *OsWAK* genes functionally analyzed, one is a negative regulator. In Arabidopsis, pectin was shown to bind AtWAK1 and AtWAK2 and to activate signal transduction [25]. Whether pectins play a similar role in rice needs to be tested. More generally, identifying the molecular signal(s) recognized by these OsWAKs proteins is now possible by the use of the mutants lines described in this study.

## Methods

### Identification of the *wak* mutants

Insertion mutants and corresponding null-segregant mutants (wild-type) were identified for the *OsWAK* genes in the OryzaTagLine mutant collection in the Nipponbare background [45, 46]. For each insertion line, PCR was used to select null mutants (siblings from homozygous line) and homozygous mutant plants in a segregating T2 family. The primers used are given in Additional file 6. These plants were allowed to self and the genotypes were confirmed by PCR in the T3 generation.

### Production of over-expresser plants

The full lengh cDNA of *OsWAK91* and *OsWAK112d* were amplified by RT-PCR using cDNA obtained from a mixture of infected and non-infected Nipponbare plants (primers in Additional file 6). The PCR products were ligated in the pCAMBIA 2300OX (kindly provided by JC Breitler, CIRAD) vector under the control of the *ubiquitin* promoter, using the BP reaction (Gateway). The inserts of the corresponding vectors were sequenced before transformation into *Agrobacterium tumefaciens* (strain EHA105). The resulting pUbi::WAK constructs were transformed into the rice embryo callus of Nipponbare, and transgenic plants were selected on plates containing 200 mg/l geneticin, 400 mg/l cefotaxine and 100 mg/l vancomycine. The primary transformants (T0 plants) were transplanted into soil and allowed to self. DNA from T0 plants was extracted for Southern blot hybridization to screen for single insertion lines. The resulting T1 single insertion lines were screened by PCR for the presence/absence of the selection marker to identify null-segregants and siblings presenting the T-DNA. These T1 plants were allowed to self and the resulting homozygous T2 seedlings were used for phenotyping upon *M. oryzae* infection.

### Fungal isolates, infection assays and chitin treatment

Rice plants and fungi were grown as described in Berruyer et al. [50] under 12 h light with temperatures between 28 and 30 °C One rice cultivar, Nipponbare (*Oryza sativa* L.) and three isolates, FR13, GY11 and CL367 of blast fungus (*Magnaporthe oryzae*) were used. The isolate CL367 is incompatible and isolates FR13 and GY11 are compatible with Nipponbare [44]. For disease phenotyping, one typical replicate was made of 4 mutant and 4 null-segregant plants grown side-by-side in 1L pots; inoculation was carried out by spraying 25 × 10^3^ conidia/mL of FR13 or GY11 isolates of *M. oryzae* (compatible strains) whereas for expression analyses we used 2 × 10^5^ conidia/mL of FR13 and CL367 conidial suspension, on fourth leaves (last fully expanded) from the bottom of 4 week old plants. All treated seedlings were placed in the dark with 100 % relative humidity for 24 h and at 20–24 °C. For mutant phenotyping, the fourth leaves were harvested and scanned at 5 days after infection for lesion observation and quantification. We differentiated susceptible lesions from resistant lesions by the presence or absence of grayish centers, indicative of sporulation.

For chitin treatment, 3 week-old Nipponbare plants (showing three fully expanded leaves) were sprayed with 0.02 % tween 20 (mock), 100 μg/mL or 1000 μg/mL of chitin solubilized in 0.02 % tween 20. The experiment was repeated four times. This chitin contains 2 to 8-mers of oligosaccharide (YSK, Yaizu Suisankagaku Industry). The third, last fully expanded leaves were harvested, frozen in liquid nitrogen, at different time points after treatment for gene expression quantifications.

### H_2_O_2_ measurements and gene expression analysis

For H_2_O_2_ measurements, leaf fragments were treated as in Vergne et al. [37] using DAB staining. Diaminobenzidine (Sigma D-8001) was solubilized to 1 mg/ml of water, excised leaves were dipped overnight (in the dark) and tissues were cleared withethanol/chloroform (4 : 1) overnight at room temperature. For quantitative RT-PCR applications, frozen tissue were ground in liquid nitrogen. Sampling was done in the growth chambers (with low light in the inoculation dark-room during the first 24 h). Each replicate was made from at least 4 plants of one given genotype that were pooled and this design was repeated three to four times. Approximately 500 μl of powder was then treated with 1 mL of TRIZOL (Invitrogen) as recommended. After a DNAse treatment (Euromedex), RNA samples (5 μg) were denaturated for 5 min at 65 °C with oligo dT_18_ (3.5 μM) and dNTP (1.5 μM). They were then subjected to reverse transcription for 60 min at 37 °C with 200 U of reverse transcriptase M-MLV (Promega, Madison, WI, USA) in the appropriate buffer. Two microlitres of cDNA (dilution 1/10) were then used for quantitative RT-PCR. Quantitative RT-PCR mixtures contained PCR buffer, dNTP (0.25 mM), MgCl_2_ (2.5 mM), forward and reverse primers (150, 300 or 600 nM final concentration), 1 U of HotGoldStar polymerase and SYBR Green PCR mix as per the manufacturer’s recommendations (Eurogentec, Seraing, Belgium). Amplification was performed as follows: 95 °C for 10 min; 40 cycles of 95 °C for 15 s, 62 °C for 1 min and 72 °C for 30 s; then 95 °C for 1 min and 55 °C for 30 s. The quantitative RT-PCR reactions were performed using a MX3000P machine (Stratagene) and data were extracted using the MX3000P software. The amount of plant RNA in each sample was normalized using actin (Os03g50890) as internal control. Gene expression was done using the measured efficiency for each gene as described in Vergne et al. [37]. The list of primers used is provided in Additional file 6.

## Additional files

Additional file 1:
**Rice WAKs regulated by blast fungus infection.** (PDF 35 kb) Additional file 2:
**Phylogenetic tree of the Arabidopsis** ***WAK*** **and EGF-containing** ***OsWAK*** **genes from rice.** The proteomes of *Arabidopsis thaliana* (TAIR release 9: 33,200 sequences) and *Oryza sativa* (TIGR Release 6.0: 67,393 sequences) were downloaded from the GreenPhyl database (http://www.greenphyl.org/cgi-bin/index.cgi) (Conte et al.*,* 2008). We retrieved *OsWAK* genes proceeding into three steps. First, we ran the hmmsearch program (Eddy, 2009) to search for kinase Hidden Markov Model (HMM) profile (PF00069.16) (Sonnhammer et al.*,* 1998) into Arabidopsis and Oryza sequences. We retrieved 3185 proteins containing a kinase motif. On this set of sequences, we again used the hmmsearch program seeking this time EGFs HMM profiles (PF00008.18, PF09120.1, PF07974.4, PF04863.4 and PF07645.6). From this second screen, we retrieved 248 proteins (33 from *Arabidopsis thaliana* and 215 from *Oryza sativa*). We extracted the kinase domain sequences of these proteins and aligned them with the E-INS-i program (default parameters) from the MAFFT website (http://mafft.cbrc.jp/alignment/software/). Based on this alignment, we generated a phylogenetic tree by the maximum likelihood method with 100 bootstrap replicates. All the genes with a *WAK* signature, explicitly containing both EGF motif(s) and kinase domain, were grouped in the tree with a bootstrap value of 86. All other genes outside this clade have been considered as outgroup. All manipulations on phylogenetic trees have been performed with the treedyn program (http://www.treedyn.org/). Empty circles: newly annotated *OsWAK* genes; black squares: *OsWAK* genes known to be differentially expressed upon infection; empty squares: *WAK* genes known to be involved in fungal resistance; asterics: *OsWAK* genes with an ACF kinase domain. (PDF 59 kb) Additional file 3:
***Wak insertion mutants***. **A.** For each *OsWAK* gene, the different splicing forms are shown as well as the position of the T-DNA insertion site. The small arrows designate the primers (Additional file 6) that were used to genotype the plants and the primers used for measuring gene expression by quantitative RT-PCR. **B.** Transcript levels for the corresponding gene as measured by quantitative RT-PCR in mutant plants (MUT) compared to the corresponding null-segregant (WT) plants. Gene expression was normalized using actin. The values represent the percent of gene expression as compared to WT (100 %); the values are the mean and standard deviation calculated from three biological replicates. (PDF 97 kb) Additional file 4:
**Disease symptoms in T0 transgenic plants over-expressing** ***OsWAK91*** **or** ***OsWAK112d.*** Unique rice T0 plants over-expressing *OsWAK91* (A, B, C; 5 lines starting with “OE”) or *OsWAK112d* (D, E, F; 6 lines starting with “OX”) were produced. Plants transformed with the empty vector are also shown (A; 4 lines starting with “EV”). The transgene expression level was normalized using Actin and is expressed as Arbitrary Unit (A, C). The values presented are unique values since each represents a unique T0 plant. Given the extremely low expression levels and thus variability in empty vectors, the very high values in over-expressor lines was considered as significant. Plants were inoculated with the virulent isolate FR13 of *Magnaporthe oryzae* and lesion number was quantified 7 dpi (B, E). Examples of symptoms are also shown (C, F). The experiments in panels A/B and C/D have not been conducted at the same time but in two separate experiments. The difference between empty vectors can thus be attributed to differences in the inoculum pressure (higher in the case of A/B). (PDF 2113 kb) Additional file 5:
**Fungal growth in OE-** ***WAK91*** **lines.** WAK91 over-expresser lines were inoculated with *M. oryzae* (GY11 moderately virulent isolate) and at the indicated time points after inoculation, the development stage of the fungus was observed under the microscope; four categories of growth stages were counted (100 interaction sites/condition). This experiment was repeated 3 times and significant differences (t-test; *P* <0.05) are shown by *. (PDF 104 kb) Additional file 6:
**Primers used for this study.** (PDF 66 kb)

## Abbreviations

DAMP: Damage Associated Molecular Pattern
EGF: Epidermal Growth Factor
MAPK: mitogen-activated protein kinases
OG: oligogalacturonide
PAMP: Pathogen Associated Molecular Pattern
PRR: pattern-recognition receptor
PTI: PAMP-triggered immunity
RLK: Receptor-like Kinase
ROS: reactive oxygen species
WAK: Wall-associated kinase
