# Supplementary material for: Several wall-associated kinases participate positively and negatively in basal defense against rice blast fungus
Source: BMC Plant Biol. 2016 Jan 16;16:17. doi: 10.1186/s12870-016-0711-x (PMC4715279; doi:10.1186/s12870-016-0711-x)
Supplement: Additional file 1: — Rice WAKs regulated by blast fungus infection. (PDF 35 kb) [file 12870_2016_711_MOESM1_ESM.pdf]

Gene	TIGR	Reference
OsWAK 14	Os02g42150	Ribot et al, 2008
OsWAK 90	Os09g38840	Ribot et al, 2008
OsWAK 91	Os09g38850	Kaku et al, 2006
OsWAK 92	Os09g38910	Ribot et al, 2008
OsWAK 112d	Os10g10130	Vergne et al, 2007
