# Supplementary figures and images for: Several wall-associated kinases participate positively and negatively in basal defense against rice blast fungus
Source: BMC Plant Biol. 2016 Jan 16;16:17. doi: 10.1186/s12870-016-0711-x (PMC4715279; doi:10.1186/s12870-016-0711-x)

Additional file 2 Delteil et al

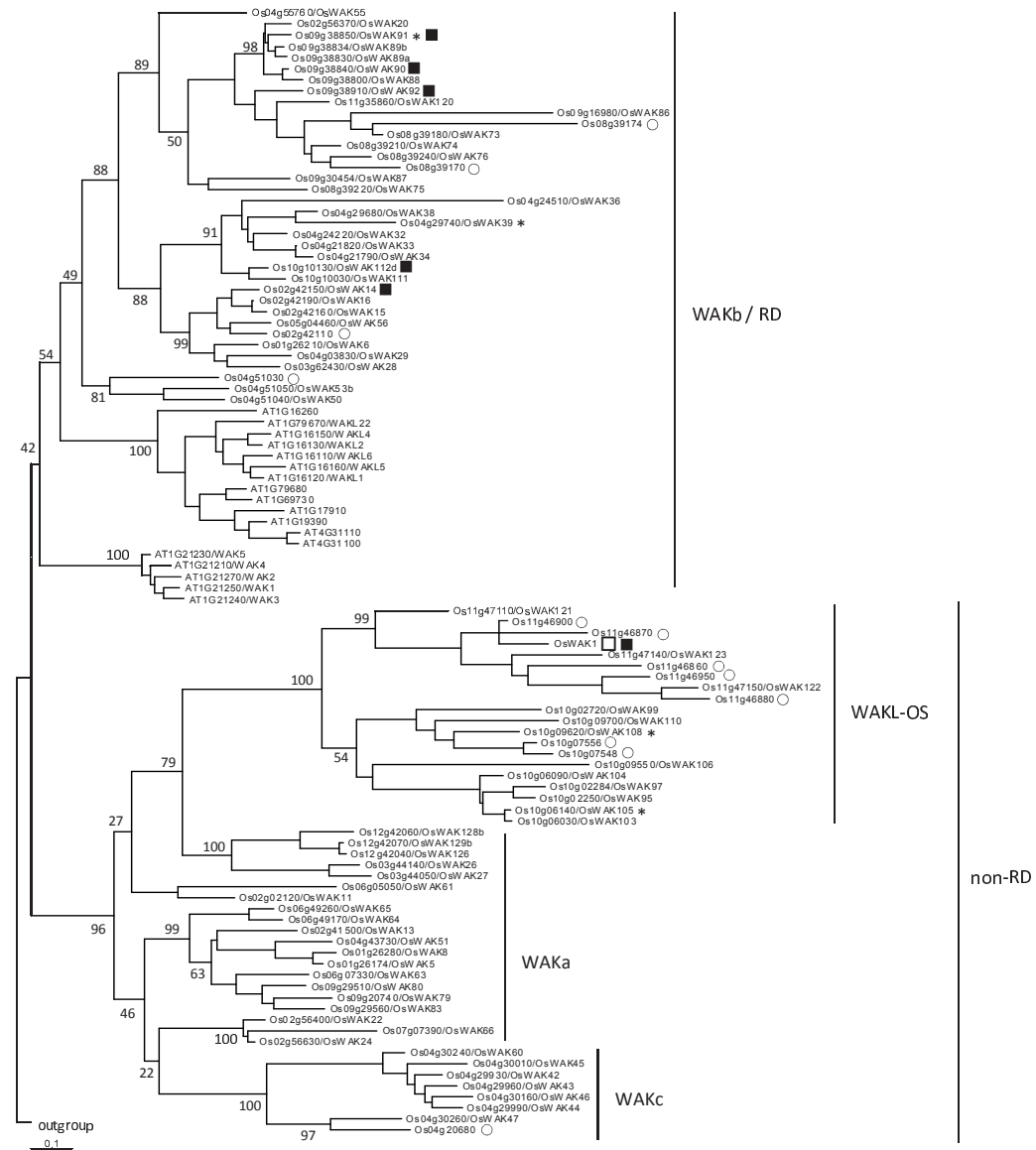

Supplement: Additional file 2: — Phylogenetic tree of the Arabidopsis WAK and EGF-containing OsWAK genes from rice. The proteomes of Arabidopsis thaliana (TAIR release 9: 33,200 sequences) and Oryza sativa (TIGR Release 6.0: 67,393 sequences) were downloaded from the GreenPhyl database (http://www.greenphyl.org/cgi-bin/index.cgi) (Conte et al., 2008). We retrieved OsWAK genes proceeding into three steps. First, we ran the hmmsearch program (Eddy, 2009) to search for kinase Hidden Markov Model (HMM) profile (PF00069.16) (Sonnhammer et al., 1998) into Arabidopsis and Oryza sequences. We retrieved 3185 proteins containing a kinase motif. On this set of sequences, we again used the hmmsearch program seeking this time EGFs HMM profiles (PF00008.18, PF09120.1, PF07974.4, PF04863.4 and PF07645.6). From this second screen, we retrieved 248 proteins (33 from Arabidopsis thaliana and 215 from Oryza sativa). We extracted the kinase domain sequences of these proteins and aligned them with the E-INS-i program (default parameters) from the MAFFT website (http://mafft.cbrc.jp/alignment/software/). Based on this alignment, we generated a phylogenetic tree by the maximum likelihood method with 100 bootstrap replicates. All the genes with a WAK signature, explicitly containing both EGF motif(s) and kinase domain, were grouped in the tree with a bootstrap value of 86. All other genes outside this clade have been considered as outgroup. All manipulations on phylogenetic trees have been performed with the treedyn program (http://www.treedyn.org/). Empty circles: newly annotated OsWAK genes; black squares: OsWAK genes known to be differentially expressed upon infection; empty squares: WAK genes known to be involved in fungal resistance; asterics: OsWAK genes with an ACF kinase domain. (PDF 59 kb) [file 12870_2016_711_MOESM2_ESM.pdf]

**A**

### *OsWAK14*

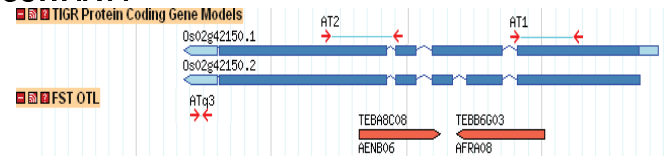

### *OsWAK91*

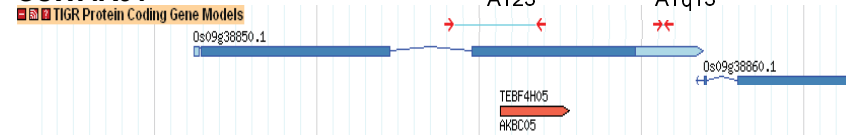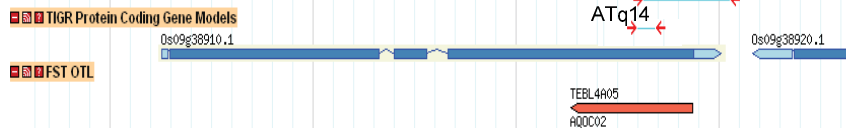

### *OsWAK112d*

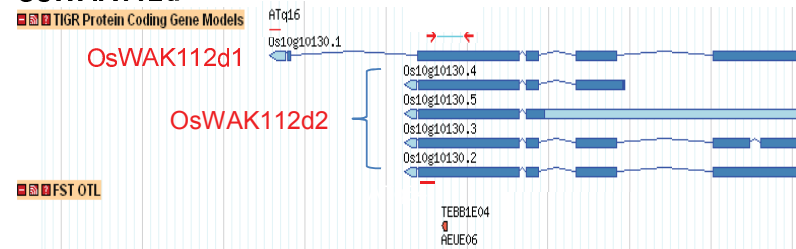

**B**

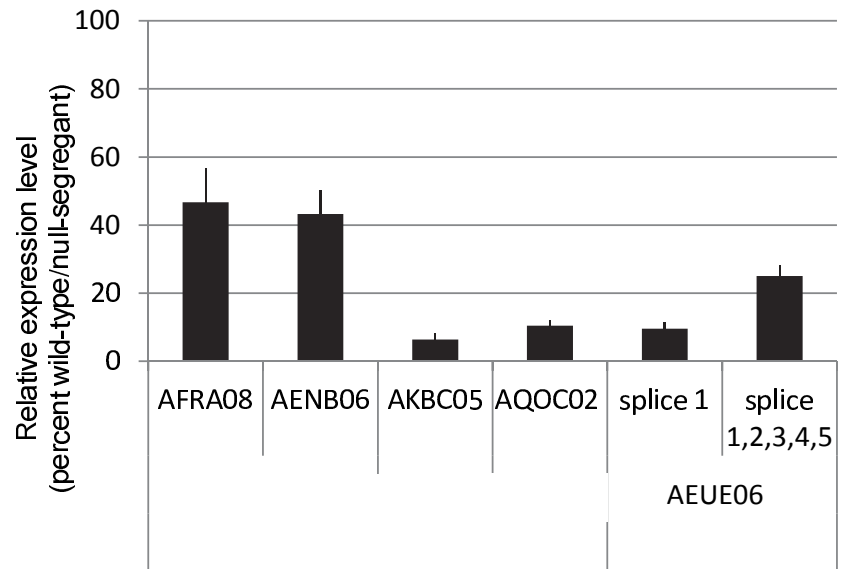

Supplement: Additional file 3: — Wak insertion mutants. A. For each OsWAK gene, the different splicing forms are shown as well as the position of the T-DNA insertion site. The small arrows designate the primers (Additional file 6) that were used to genotype the plants and the primers used for measuring gene expression by quantitative RT-PCR. B. Transcript levels for the corresponding gene as measured by quantitative RT-PCR in mutant plants (MUT) compared to the corresponding null-segregant (WT) plants. Gene expression was normalized using actin. The values represent the percent of gene expression as compared to WT (100 %); the values are the mean and standard deviation calculated from three biological replicates. (PDF 97 kb) [file 12870_2016_711_MOESM3_ESM.pdf]

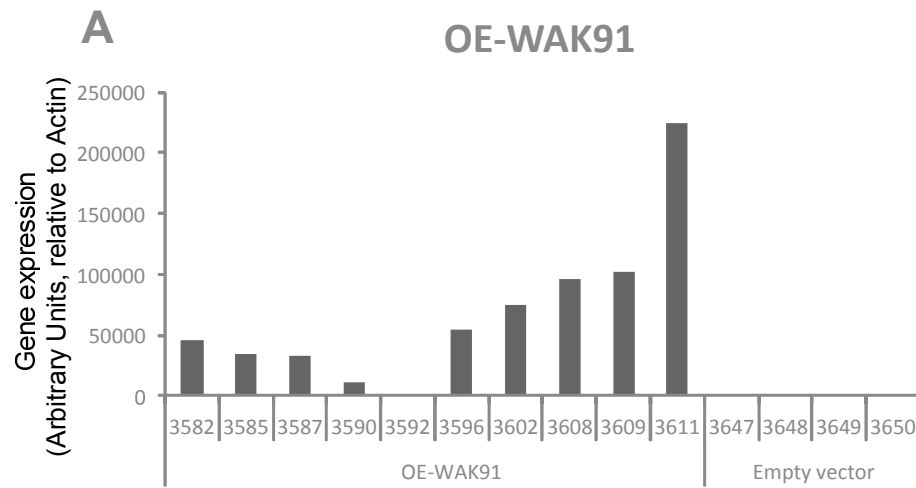

**B**

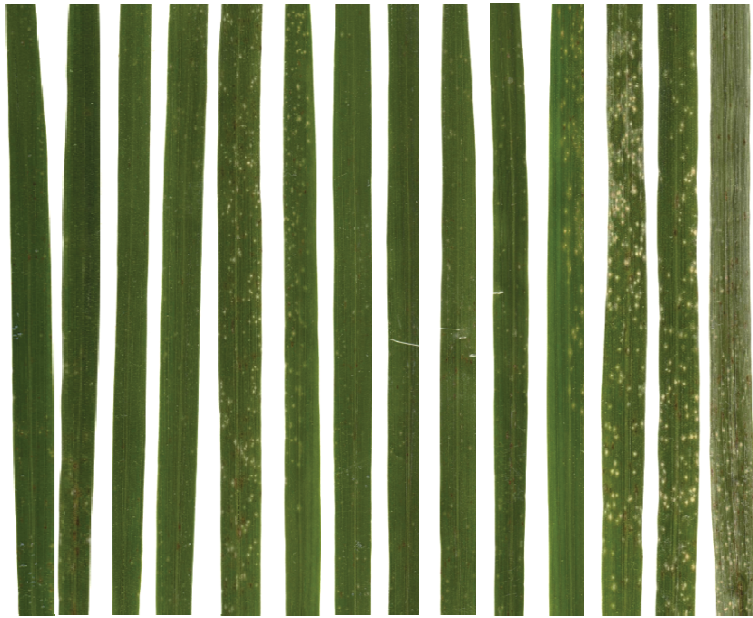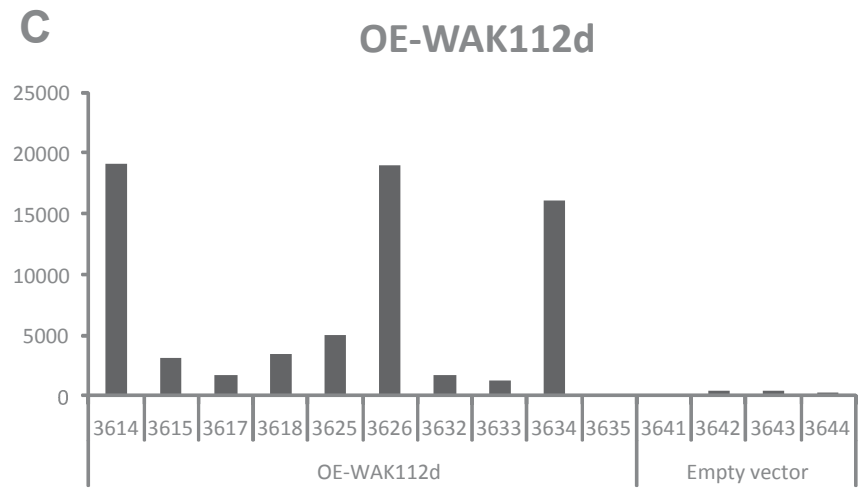

**D**

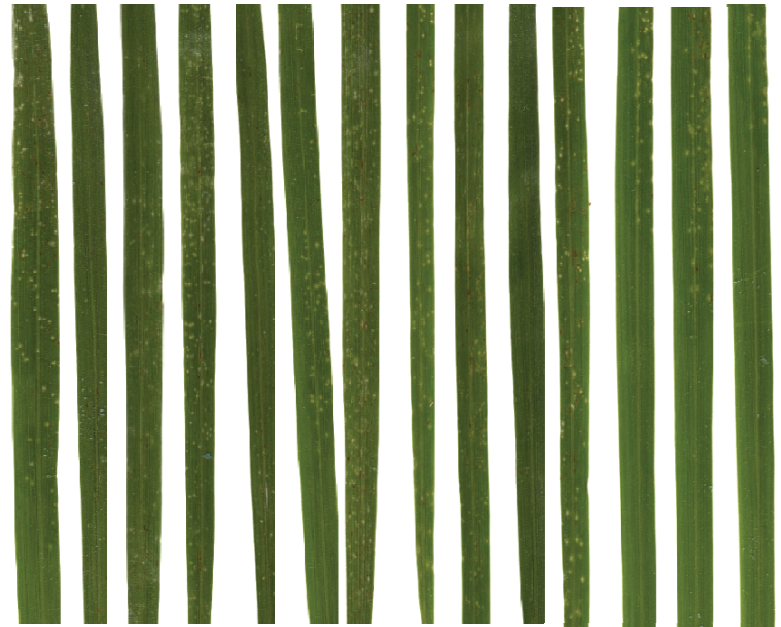

Supplement: Additional file 4: — Disease symptoms in T0 transgenic plants over-expressing OsWAK91 or OsWAK112d. Unique rice T0 plants over-expressing OsWAK91 (A, B, C; 5 lines starting with “OE”) or OsWAK112d (D, E, F; 6 lines starting with “OX”) were produced. Plants transformed with the empty vector are also shown (A; 4 lines starting with “EV”). The transgene expression level was normalized using Actin and is expressed as Arbitrary Unit (A, C). The values presented are unique values since each represents a unique T0 plant. Given the extremely low expression levels and thus variability in empty vectors, the very high values in over-expressor lines was considered as significant. Plants were inoculated with the virulent isolate FR13 of Magnaporthe oryzae and lesion number was quantified 7 dpi (B, E). Examples of symptoms are also shown (C, F). The experiments in panels A/B and C/D have not been conducted at the same time but in two separate experiments. The difference between empty vectors can thus be attributed to differences in the inoculum pressure (higher in the case of A/B). (PDF 2113 kb) [file 12870_2016_711_MOESM4_ESM.pdf]

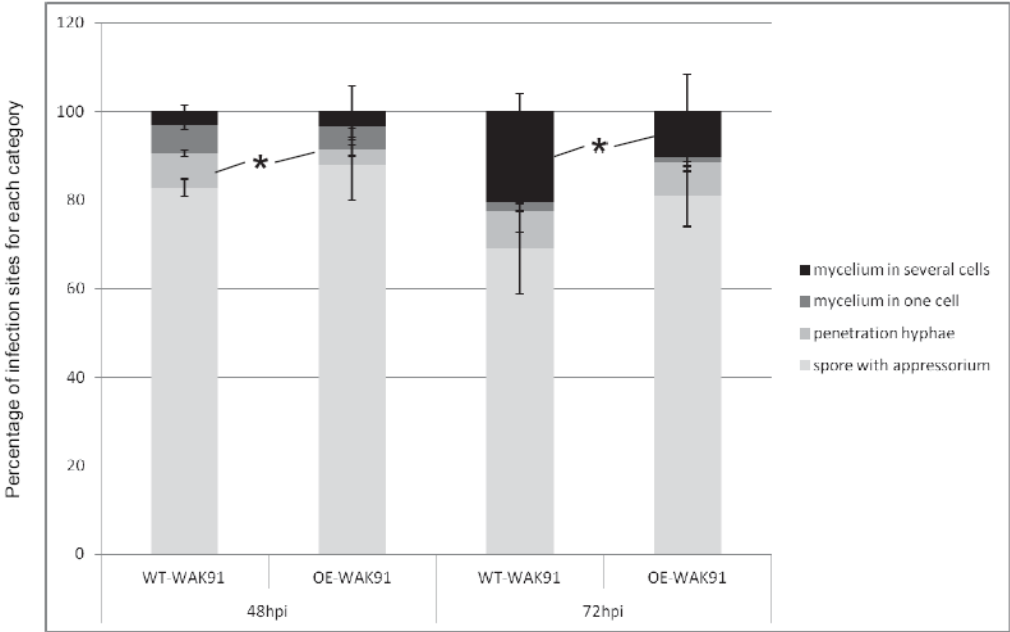

Supplement: Additional file 5: — Fungal growth in OE- WAK91 lines. WAK91 over-expresser lines were inoculated with M. oryzae (GY11 moderately virulent isolate) and at the indicated time points after inoculation, the development stage of the fungus was observed under the microscope; four categories of growth stages were counted (100 interaction sites/condition). This experiment was repeated 3 times and significant differences (t-test; P <0.05) are shown by *. (PDF 104 kb) [file 12870_2016_711_MOESM5_ESM.pdf]
