# Supplementary material for: Several wall-associated kinases participate positively and negatively in basal defense against rice blast fungus
Source: BMC Plant Biol. 2016 Jan 16;16:17. doi: 10.1186/s12870-016-0711-x (PMC4715279; doi:10.1186/s12870-016-0711-x)
Supplement: Additional file 6: — Primers used for this study. (PDF 66 kb) [file 12870_2016_711_MOESM6_ESM.pdf]

Additional file 6 Delteil et al

Type	Primer	Gene Name	Comment	Forward	Reverse
qRT-PCR	33	Os03g50885	actin, putative, expressed	GCGTGGACAAAGTTTTCAACCG	TCTGGTACCCTCATCAGGCATC
	PBZ1	Os12g36880	PBZ1 pathogenesis-related Bet v I family protein, putative, expressed	AGGCATCAGTGGTCAGTAGAG	CGGGTCTTGTATGTGCTTCC
	CHI7	Os06g51050	CHIT7 - Chitinase family protein precursor, expressed	CCGCCTTCCTAGTTGATATTTG	GTCTCACAATCTCGTGTGCATT
	PAL	Os02g41680	PAL phenylalanine ammonia-lyase, putative, expressed	CGTGCGGTGTTGTTTTTATC	GCTTTTGAAACCTGCCACTC
	osMT2b	Os05g02070	OsMT2b Metallothionein-like protein 2C	ACCGTCGTCGTCGTTGTCAG	GCATGAGGAGATGGAGCAGGAG
	SPL7	Os05g45410	SPL7 HSF-type DNA-binding domain containing protein, expressed	CGGATTAGAGGCTTGCGTGTTAC	GCACAGTAGTCAGCGGATAGAAC
	ATq3	Os02g42150	OsWAK14 - OsWAK receptor-like protein kinase, expressed	CCGTTCTGAACAGGGTATGC	CTTTGCCACCTGGCCTTAAT
	ATq12	Os09g38840	OsWAK90 - OsWAK receptor-like protein kinase, expressed	TTTCTTGTGTGAGTTTGGATCG	CTACGTGCGTATCATACATGGAG
	ATq13	Os09g38850	OsWAK91 - OsWAK receptor-like protein kinase, expressed	CCCGGTAAGTGAGGGTACAA	TGATCCAAAACCACTCCAGA
	ATq14	Os09g38910	OsWAK92 - OsWAK receptor-like protein kinase, expressed	ACCTGCAAATCATGGAGGAG	TCAAGTGCCATCTCAACCTG
	ATq16	Os10g10130.1	OsWAK112d1	TCGCAATACCCGGATTTAGT	TTAGGATCCCAAATCGCTTG
	ATq39.1	Os09g38850	OE-WAK91	TGAGGTAGGTGGAGATGACACTCG	CCACACCATGCTCTTGCTGC
	ATq41	Os10g10130	OE-WAK112	TTGCAAGCATGACAGCGGTTAC	AACCCTCTTCAAGGCCAAACGG
genotyping	AT1	Os02g42150	*WAK14-1* mutant (AFRA08)	TATTTTGCTTCGTTTTGGGG	TCGATGCTACCTTGTTCGAG
	AT2	Os02g42150	*WAK14-2* mutant (AENB06)	GAAGGTGGTCATGCAGTGTG	TTACATCAGCAAAGCAACGC
	AT23	Os09g38850	*WAK91-1* mutant (AKBC05)	GCACCAACTGCTCTGTTTCA	GCAATGGCACTTCTGTCTCA
	AT4	Os09g38910	*WAK92-1* mutant (AKOC02)	ACCTGCAAATCATGGAGGAG	GGCCATGTCGTCAGTACAAA
	AT5	Os10g10130	*WAK112d -1* mutant (AEUE06)	GGTCACTGGCTCCTTCAGTC	CATCTTCCACCGTGATGTGA
cloning	AT-FL12	Os10g10130	OsWAK112d	GATGCTTAGCTAGACATGC	GGATAAACACATCTACCGTGG
	AT-FL14	Os09g38850	OsWAK91	TCATTCAACCACCGGCTATG	CCACACCATGCTCTTGCTGC
